# Dislodgment Effects of Different Cage Arrangements in Posterior Lumbar Interbody Fusion: A Finite Element Study

**DOI:** 10.3390/bioengineering11060558

**Published:** 2024-05-31

**Authors:** Shih-Chieh Yang, Chih-Lin Wu, Yuan-Kun Tu, Pao-Hsin Liu

**Affiliations:** 1 Department of Orthopedic Surgery, E-Da Hospital, No. 1, Yida Rd., Jiaosu Village Yanchao District, Kaohsiung City 82445, Taiwan; edaspine@gmail.com (S.-C.Y.); ed100130@edah.org.tw (Y.-K.T.); 2 Department of Biomedical Engineering, I-Shou University, No. 8, Yida Rd., Jiaosu Village Yanchao District, Kaohsiung City 82445, Taiwan; p88881035@gmail.com

**Keywords:** cage dislodgment, fibula autograft, biomechanics, posterior lumbar interbody fusion, cage arrangement

## Abstract

The vertebral cage has been widely used in posterior lumbar interbody fusion. The risk of cage dislodgment is high for patients undergoing lumbar fusion surgery. Therefore, the main objective of this study was to use a lumbar fusion model to investigate the effects of cage dislodgment on different cage arrangements after PLIF. Finite element analysis was used to compare three PEEK cage placements, together with the fibula-type cage, with respect to the four kinds of lumbar movements. The results revealed that a horizontal cage arrangement could provide a better ability to resist cage dislodgment. Overall lumbar flexion movements were confirmed to produce a greater amount of cage slip than the other three lumbar movements. The lower part of the lumbar fusion segment could create a greater amount of cage dislodgment for all of the lumbar movements. Using an autograft with a fibula as a vertebral cage cannot effectively reduce cage dislodgment. Considering the maximum movement type in lumbar flexion, we suggest that a horizontal arrangement of the PEEK cage might be considered when a single PEEK cage is placed in the fusion segment, as doing so can effectively reduce the extent of cage dislodgment.

## 1. Introduction

Since the clinical introduction and first application of the interbody fusion technique, posterior lumbar interbody fusion (PLIF) combined with pedicle screw fixation has been an option for the treatment of lower back pain caused by severe disc degeneration, vertebral instability, or spondylolisthesis. The technical improvements in and favorable surgical outcomes of PLIF make this procedure a popular choice among spine surgeons [1,2,3,4]. However, adverse surgical outcomes resulting from PLIF surgery are clinically notable [5,6]. Most surgical failures are due to postoperative cage dislodgment, the destruction of adjacent intervertebral discs, the degeneration of adjacent facet joints, and/or vertebral cage subsidence [7,8]. Disc dislodgment will lead to serious nerve compression and peripheral tissue damage, while cage subsidence can result in the instability of the vertebral fusion segment [9,10,11]. Previous studies have also indicated that the use of a vertebral cage for interbody fusion is common and has a high fusion rate; however, 1.4–2.2% of patients may experience complications such as cage subsidence, cage dislodgment, temporal neural injury, and adjacent disc degeneration [12,13]. The use of vertebral cage fixation techniques, such as PLIF, allows patients to regain their intervertebral height and increases the stability of the spinal segments [14]. Conventional PLIF usually requires the insertion of two cages for parallel placement, but this surgical selection would require a wider laminectomy, causing more surgical trauma and carrying a higher risk of neurological injury [15,16]. Results regarding the traditional clinical application of PLIF using two intervertebral cages have been widely reported [17]. Using two cages for vertebral interbody fusion generates greater medical costs in clinics. In vitro biomechanical studies have indicated that a traditional vertebral single-cage approach to PLIF with unilateral facet-fixed screws also provides sufficient fusion stability with respect to the interbody segment [18]. In recent years, in vitro studies have also shown that the oblique insertion of a single vertebral cage in PLIF could reduce cage exposure, allow the cage to be inserted accurately in the appropriate position, and significantly reduce the cost of surgical treatment [19]. Although a growing number of studies have indicated that a single vertebral cage can be successfully used in PLIF surgery for patients with disc failures [20], to the best of our knowledge, there are no studies investigating the biomechanical influences of employing different insert positions when using a single-cage technique for PLIF in comparison. Current studies have also found that PLIF surgery involving a single vertebral cage can also adequately reflect the consequences of fusion with respect to providing a stable fusion segment [9,21]. Previous studies have also shown that the placement of a single oblique cage could ensure excellent stability of the disc and facet joints between the upper and lower vertebrae, and the post-treatment results were similar to those for the effects of double-cage insertion [22,23]. However, the biomechanical effects of different insertion arrangements for a single cage have not been investigated; more precisely, the relationship between different cage placements and cage dislodgment has not been examined in previous studies. On the other hand, another fusion technique—namely, autologous bone grafting—has also been applied to replace the role of the traditional cage, potentially facilitating a more rapid interbody fusion.

Since 1992, three-dimensional numerical models have been used for the investigation of spinal projects and surgical evaluations [24]. Finite element analysis offers advantages such as allowing the quick and easy construction of the cage structure and the modification of the geometry of the designed cage to evaluate size and shape effects [25,26]. On the other hand, finite element analysis does not require the use of a human cadaver or animal samples. Finite element analysis has been widely used for biomechanical studies and has been applied successfully in many lumbar spine studies. The biomechanical effects of the different arrangements of single-cage placement in PLIF are not clear, especially regarding the effects of cage dislodgment. Therefore, the purpose of this study was to investigate and compare cage dislodgment in different arrangements with a single PEEK cage in PLIF treatment. In addition, in order to understand whether the autograft (fibular block) is sufficient for fixed stability after PLIF, we also performed a biomechanical evaluation of lumbar fusion using a finite element model to compare the cage dislodgment influences between the PEEK cage and the autograft bone cage in PLIF.

## 2. Materials and Methods

**Figure 1:**
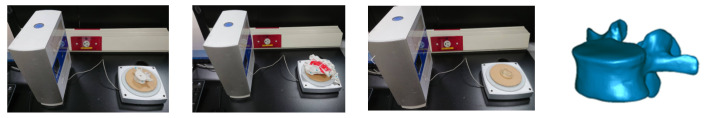
Spine model reconstruction using the reverse engineering technique. Laser scanning was applied to obtain the geometric features of the vertebrae, sacrum, and discs (from left to right). A surface model, such as of the lumbar vertebra, was reconstructed to reflect the real geometry of the vertebral shape. All of the surface models, including the vertebrae, sacrum, and discs, were further aligned according to the spinal anatomic position.

The spine model, which consisted of a section of the spine ranging from the 12th segment of the thoracic vertebrae to the whole sacrum, was reconstructed based on a reverse engineering (RE) technique through scanning a standard anatomical model of artificial medical-trained aids developed by NEXTENGINE (Nextengine Inc., Santa Monica, CA, USA). The selected version of the artificial spine model can reduce variations in different human subjects with respect to vertebral size and shape, as well as the spinal curve of vertebral alignment, in order to reveal the analytical influences of parameter combinations during surgical reconstruction. Each vertebra (including the sacrum) and disc were individually scanned to obtain surface geometries of the models and perform spinal vertebral alignment according to the anatomical position and lumbar segmental curve (Figure 1). The main reason for this is that the purpose of this study was to investigate the cage dislodgment effects of different PEEK cage arrangements and the fibular bone cage rather than to examine biomechanical behaviors of the lumbar segment relating to the inner bone structure. Moreover, the lumbar spine replica in this study was a very real geometric acrylic model, with an actual vertebral anatomy and a disc structure. Therefore, the laser-scan-based numerical model of the lumbar vertebrae and discs did not reduce the accuracy of the lumbar spinal geometry. The relevant literature for the RE-scan-based model for spinal investigation was also used for finite element analysis [27]. In the RE-scan-based model, the cortical bone layer thickness was assumed to have a uniform thickness, which was a simplified setting not requiring a CT scan. Hence, the heterogeneous cortical bone layer was reconstructed via a CAD process. This study mainly focuses on the influence of cage dislodgment under four lumbar movements. Consequently, the FE model of the artificial lumbar replica could be reconstructed via the RE scanning technique to investigate the cage dislodgment in PLIF.

**Figure 2:**
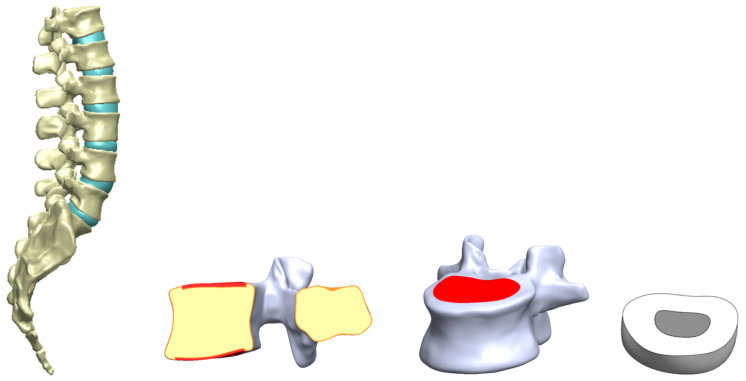
The intact spinal model, which included two materials and structures of cortical layer and cancellous bones, consisted of thoracic and lumbar vertebrae, the sacrum, and discs. The endplate was also reconstructed using the CAD procedure at the superior and inferior vertebral bodies (with a 1 mm thickness). The annulus fibrosus of the disc model (the right-side model) was assumed to have a solid volume in the finite element model. The structure of the nucleus pulposus was also reconstructed to provide a real numerical model of the lumbar spine.

The standard RE-based model of the spine was further transferred into the Geomagic Essentials (Geomagic Inc., Morrisville, NC, USA) software product to apply a surface-optimized process and thus generate a solid model including hard tissues of the vertebrae and soft tissues of the intervertebral discs. The cortical thickness of the spinal solid model was considered to be in the 0.38 (±0.06) mm range, as specified in previous studies [28]. The structure of the endplate, with a 1 mm thickness, was reconstructed using a CAD procedure to reflect the real structure within the inferior and superior surfaces of vertebral body. The complete thoracic–lumbosacral model, which was contained within hard and soft tissues, was reconstructed as a standard model to provide data for investigating the effects of cage dislodgment via finite element analysis (FEA) (Figure 2).

**Figure 3:**
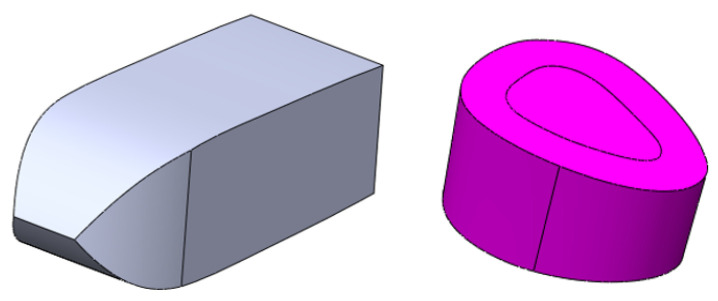
The two types of cages used to investigate the effects of cage dislodgment: the PEEK material (the bullet-shaped cage) and the fibular block (the cylinder-shaped cage).

A bony resection of the unilateral lumbar facet joint of the spinal model was executed to provide an entrance for inserting the cage and fibular graft. Two types of cages were evaluated to compare the biomechanical effects of cage dislodgment. One of the cage models, which was a bullet-shaped cage, was reconstructed through CAD design in terms of its size and shape according to a commercialized product to facilitate a stability evaluation in the finite element analysis. Another cage model, which was selected from a fibular block and designed as a cylinder-shaped cage, was also chosen according to clinic experience to evaluate the cage’s function with different shapes and materials. The bullet-shaped cages were 12.0 mm in length in the L1–L3 vertebrae and 16.0 mm in length in the L4–L5 vertebrae; moreover, their width and height were 7.0 mm and 6.5 mm, respectively. The height of the cylinder-shaped cage was 6.5 mm (Figure 3). The pedicle fixation system, which was affixed to increase the stability of cage insertion and thus decrease cage dislodgment after lumbar movements, consisted of four pedicles and two rods, providing a rigid structure for cage positioning between two vertebrae according to different fusion segment positions. The diameters of the pedicle screw and rod were 6.5 and 6.0 mm, respectively.

**Figure 4:**
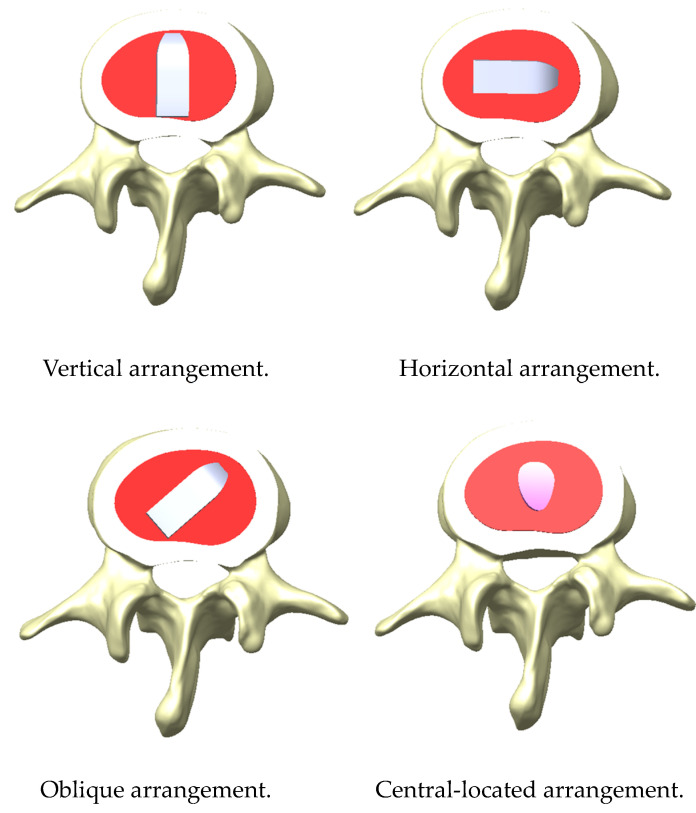
The three cage arrangements, namely, vertical, horizontal, and oblique, for the bullet-shaped cage. The cylinder-shaped cage arrangement entailed placement in the central region.

Three implantation arrangements for placing the bullet-shaped cages between two vertebrae were selected—namely, anteroposterior placement (vertical arrangement), mediolateral placement (horizontal arrangement), and 45° oblique placement (the oblique arrangement)—to investigate the effects of anti-slip ability in different cage arrangements. The cylinder-shaped cage was the only arrangement in which a fibular block was placed in the central area between two vertebrae (Figure 4).

The boundary condition was fixed at the surfaces of the bilateral alar joint in the hip. The material properties of the FEA were set according to previous studies [29,30] for the cortex, cancellous bone, PEEK cage, fibular cage, pedicle screws, rods, endplates, discs, facet joint cartilages, and ligaments (Table 1). The number and distribution of spinal ligaments are very complex, and model reconstruction during finite element analysis, particularly in the ligament soft tissues, could not be completed according to the size and shape of the real ligaments. Therefore, the finite element analysis simplified the ligament structures. In general, the ligaments were assumed to be spring or truss elements and a point distribution of ligament positions was assumed. This study also used a simplified ligament assumption to simulate the effects of the ligament structure as much as possible. Therefore, a ligament was replaced by a single truss element located at two points between vertebrae like in this study. The ligaments were also positioned with a single truss element by following previous studies [31,32]. Therefore, six ligaments—namely, the anterior longitudinal ligament (ALL), posterior longitudinal ligament (PLL), ligament flava (LF), interspinal ligament (ISL), supraspinal ligament (SSL), and intertransverse ligament (ITL)—were included in the FE models. All types of ligaments were assumed to be truss elements and to have tension-only properties. 

**Figure 5:**
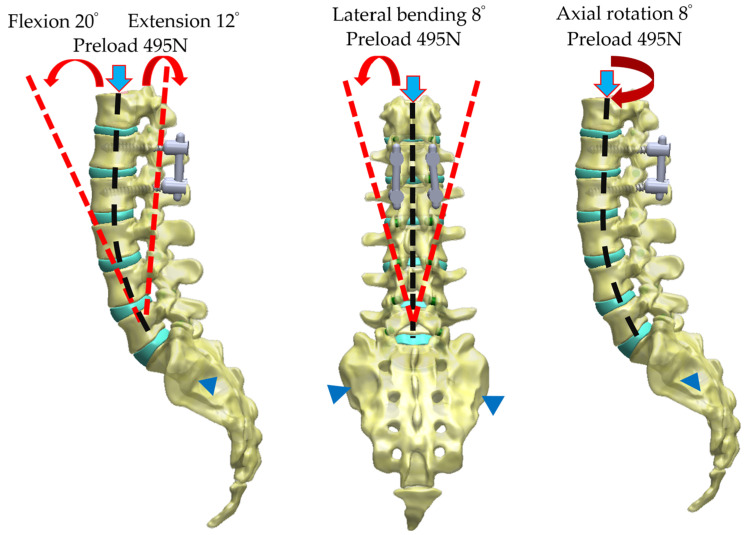
The lumbar activities of flexion, extension, lateral bending, and axial rotation were applied for testing at angles of 20°, 12°, 8°, and 8°, respectively. The 495 N preload was also referred to as a follower load along the curvature of the spine during lumbar movement testing [36,37]. The boundary condition was fixed at the region of the alar joint on the sacrum.

The loading conditions ensured the application of lumbar movements to evaluate cage dislodgment after posterior lumbar interbody fusion surgery. The lumbar activity of flexion was studied from 0° to 20° in this study, and each step size of increased flexion was 10°. The appropriate range of motion in lumbar flexion was selected as 20° (7.5 Nm), as the maximum amount of lumbar flexion in humans is 25° [33]. To investigate the maximum effect of cage dislodgment, major flexion activity was evaluated first; moreover, major cage dislodgment was detected and confirmed in the flexion of lumbar movement in previous studies [34,35]. Furthermore, as this study was also conducted to provide a comprehensive understanding of cage dislodgment, the other three lumbar movements were still analyzed. The lumbar activities of extension, lateral bending, and axial rotation in these fusion models were also executed at the angles of 12°, 8°, and 8°, respectively (Figure 5). A preload of 495 N of body weight was applied to the superior surface of the thoracic vertebra to account for the effects of weight bearing. The preload was chosen according to the percentage of body weight of a normal male adult [36,37].

**Figure 6:**
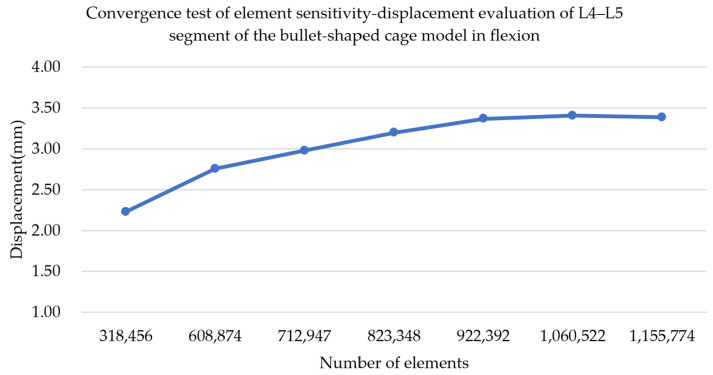
The tendency of the convergence test in the displacement of L4–L5 fusion segment through 10° flexion.

Finite element analysis has long been used to explore the topic of clinical surgical reconstruction or biomechanical evaluation. The results of finite element analysis have also been proven to have a certain reference value and credibility in previous studies [23,34]. The results of finite element analysis could provide numerical trends according to different mechanical indices as a research reference. To evaluate whether the results of the finite element analysis were reliable, a convergent test with element number sensitivity was performed before the formal analysis. We believe that the FE results of the study could provide an improved, reliable reference if the convergence results meet the convergence criterion of 5% or below [38]. The element sensitivity of the convergence test was determined to evaluate the mesh sensitivity in the displacement of the cage, which was evaluated at 10° flexion in the lumbar model of the bullet-shaped cage. The convergence test was considered to have stopped increasing the elements when the value of percent relative error (PRR) was less than 5%. The displacement of the L4–L5 fusion segment during the flexion test with the bullet-shaped cage reached the stop criterion at element numbers of 1,060,522 approximately through to the element convergence with mesh sensitivity. The model of the cylinder-shaped cage in the L4–L5 segment was also evaluated via a convergence test. To verify the convergence performance, the results of the convergence test for the model of the bullet-shaped cage at 10° degrees of lumbar flexion were plotted and are shown in Figure 6. Furthermore, the element numbers—which were confirmed to be 1,041,484 and 1,060,522 for cylinder- and bullet-shaped cage models, respectively—were evaluated through convergent testing of element sensitivity according to a less-than-convergence criterion of 5%. The convergence criterion for this finite element analytical model was reached, providing reliable results of cage dislodgment. The percent relative error (PRR) was determined according to PRR = ((current approximation − previous approximation)/current approximation) × 100%.

**Figure 7:**
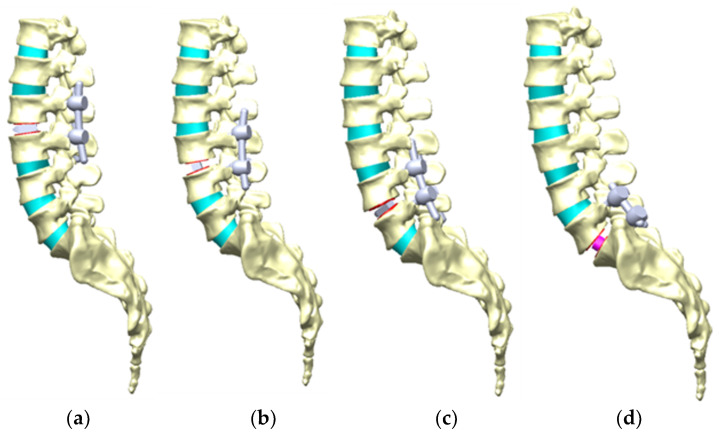
Different locations of one-level cage fusion between two vertebrae. For example, (**a**) D2: between lumbar 2 and lumbar 3 with a PEEK cage; (**b**) D3: between lumbar 3 and lumbar 4 with a PEEK cage; (**c**) D4: between lumbar 4 and lumbar 5 with a PEEK cage; (**d**) D5: between lumbar 5 and the sacrum with a fibular cage.

One-level fusion with two types of cages was located at five positions from top to bottom, encompassing L1–L2 (D1), L2–L3 (D2), L3–L4 (D3), L4–L5 (D4), and L5–S1 (D5) (Figure 7). Therefore, to compare the two types of cages and three types of cage arrangements, we used 20 FEA models for each lumbar movement. In total, 80 FEA models were evaluated to investigate the effects of cage dislodgment in the different fusion segments after PLIF.

**Table 1:** The material properties of the finite element lumbar fusion model [29,30,39,40,41].

	Young’s Modulus (MPa)	Poisson’s Ratio	Cross-Sectional Area (mm)
Ti6Al4V (pedicle and rod)	110,000	0.3	
Vertebral cortical bone	12,000	0.3	
Vertebral cancellous bone	100	0.3	
Disc	450	0.35	
Fibular cortical bone	13,700	0.3	
Fibular cancellous bone	700	0.3	
PEEK cage	3500	0.3	
End plate	12,000	0.32	
Facet joint cartilage	23	0.32	
ALL	20	0.3	63.7
PLL	20	0.3	20
LF	19.5	0.3	40
ISL	11.6	0.3	40
SSL	15	0.3	30
ITL	58.7	0.3	3.6

(ALL = anterior longitudinal ligament; PLL = posterior longitudinal ligament; LF = ligament flava; ISL = interspinal ligament; SSL = supraspinal ligament; ITL = intertransverse ligament).

A validation of this study was carried out to examine the reliability of the finite element (FE) model by measuring the intact lumbar spine motion to compare it with real human subjects. The range of motion (ROM) in the lumbar spine was evaluated from lumbar L1 to the sacrum in order to compare it with previous human studies. To reflect the intact lumbar motion of a real human structure, the FE model of the intact spine consisted of the thoracic spine (T12), lumbar vertebrae (L1–L5), sacra (S1–S5), the coccyx, hips, intervertebral discs, and spinal ligaments. Alar joints between the hip and sacrum were also created to provide a complete intact spine model with the ability to move. Previous studies were selected to compare with the present FE study to confirm the reliability of the numerical FE model in order to provide further quantitative investigations of cage dislodgment after PLIF [42,43,44]. The comparisons of the ROM for each lumbar motion are shown in Table 2. The comparison found a good agreement between the present FEA results and real human data through the ROM of lumbar movements. The ROMs of the intact lumbar spine of the standard FE model were demonstrated to be located between human measurement values when compared with real human subjects. In other words, the validation confirmed the reliability of the present FE model. Therefore, the results on the cage dislodgment of the FEA could be used to evaluate the effects of different cage arrangements in PLIF surgery.

## 3. Results

**Figure 8:**
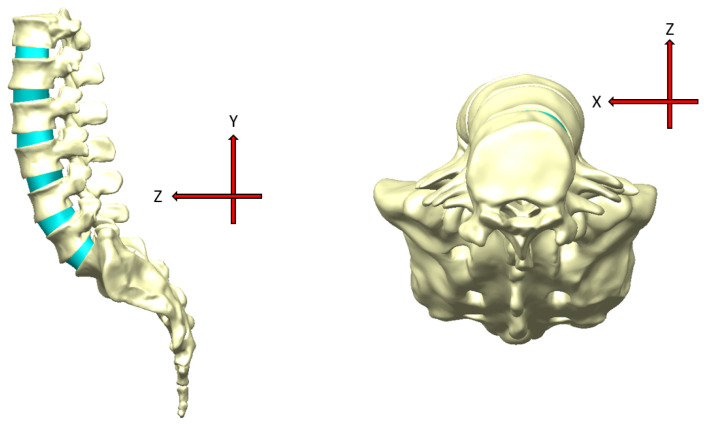
The coordinate system of the finite element lumbar fusion model was defined to evaluate cage dislodgment during four lumbar movements. The negative Z direction is represented as the cage dislodgment direction to calculate the slippage distance between two fusion vertebrae.

**Figure 9:**
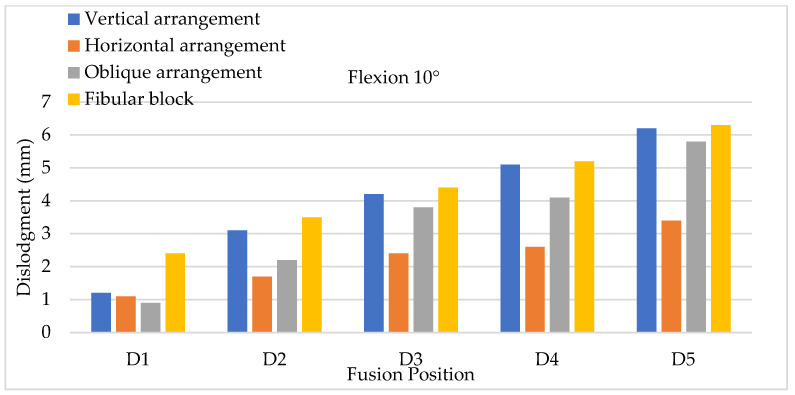
Flexion testing using a 10° angle to evaluate cage dislodgment in different fusion locations from top to bottom.

**Figure 10:**
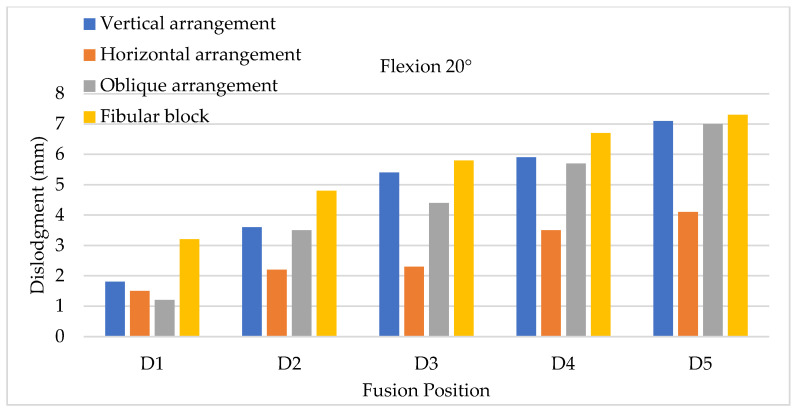
Flexion testing with a 20° angle to evaluate cage dislodgment in different fusion locations from top to bottom.

The X-Z plane (the transverse plane) was set as the reference plane for evaluating cage dislodgment between two vertebrae. The positive Z axis represents the anterior direction of the lumbar model according to the corresponding anatomic definition, and the X axis represents the mediolateral direction of the lumbar model (Figure 8). Therefore, cage dislodgment was calculated as the displacement of the cage centroid before and after flexion in this plane. The evaluation of the backward slippage of the cage (cage displacement with respect to the negative Z direction) under 10° flexion from top to bottom (five positions) on the X-Z plane significantly showed that an increasing tendency for cage dislodgment was detected at the lower positions of the lumbar spine when two positions with adjacent disc segments were compared (Figure 9). In other words, a lower placement of the inserted cages could produce a greater amount of backward slip; therefore, a high risk of cage dislodgment was discovered by the FE models of lower cage insertion in posterior lumbar interbody fusion. The maximum and minimum cage dislodgment was clearly identified at the positions of D5 and D1, respectively. Both the bullet-shaped and cylinder-shaped cages were confirmed to exhibit the same tendency, wherein there was a greater cage displacement, reflected in the lower cage position. Furthermore, all the cage arrangements for the bullet-shaped cage, such as vertical, horizontal, and oblique arrangements, showed the same situation regarding cage dislodgment. The specific PEEK bullet-shaped cage in this study had a similar dislodgment pattern, which indicates that a lower fusion can reflect a greater amount of cage displacement. The trends in the cage dislodgment for lower fusion were not applicable to all types of bullet-shaped cages due to different cage designs. The fibular cage—a cylinder-shaped structure—was observed to have the worst cage dislodgment for all fusion segments compared with the total cage arrangements under flexion movements. Excluding the fibular cage, the maximum cage dislodgment was observed in the vertical cage arrangement for flexions of 10° and 20°. Furthermore, a comparison of the three types of cage arrangements for the bullet-shape cage indicated that the minimum cage displacements of the oblique arrangement were only detected in the position of D1, and those of the horizontal arrangement were discovered at the D2, D3, D4, and D5 positions. Obviously, cage dislodgment was prevented in horizontal cage insertion for most fusion segments in posterior lumbar interbody fusion. When further evaluating lumbar flexion of 20°, the results regarding cage dislodgment showed that the increased tendency of cage backward displacement was confirmed to cause a higher risk of cage dislodgment with more than a 10° movement under flexion activity after posterior lumbar interbody fusion (Figure 10). Moreover, the lowest amount of cage dislodgment was still detected in the horizontal cage arrangement, reflecting more effective cage insertion, except in the D1 position. The maximum and minimum cage dislodgments for all the types of cages were located at the D5 and D1 positions, respectively. Obviously, the fibular (cylinder-shaped) cage was observed to be insufficient for avoiding cage dislodgment in different fusion positions. The maximum amount of backward cage dislodgment was clearly observed with backward sliding in flexion movements. FEA revealed that the horizontal cage arrangement’s ability to significantly prevent sliding could reduce the cage dislodgment between two vertebrae at flexions of both 10° and 20°. Therefore, the effects of the three types of PEEK cage arrangements indicated that the best performance was exhibited by the horizontal cage arrangement in the close positions in two flexion stages, and the worst performance was exhibited by vertical and oblique cage arrangements in positions D2 to D5. Further comparison of the vertical and oblique cage arrangements revealed that the worst case was the oblique cage arrangement in two conditions of lower fusion positions (such as D3, D4, and D5) and higher flexion angles (such as 20°). The larger flexion angles of lumbar movement reflected greater cage dislodgments in all types of cages and in all positions of the cage fusion segments under flexion movements.

**Figure 11:**
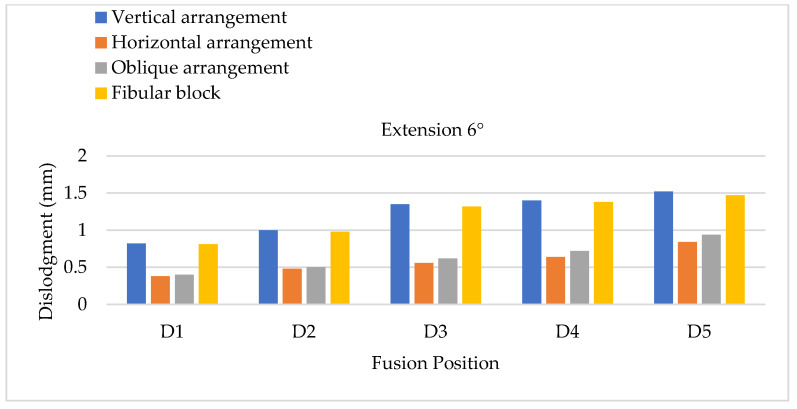
Extension testing using 6° extension to evaluate cage dislodgment in different fusion locations from top to bottom.

**Figure 12:**
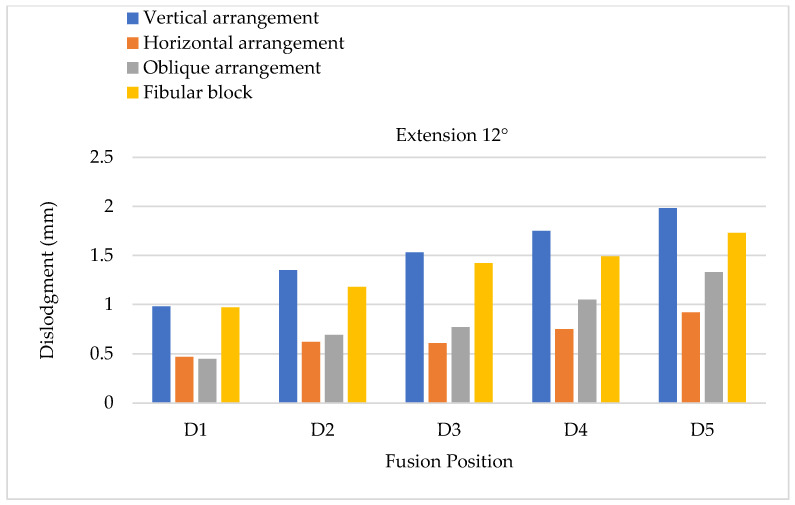
Extension testing using 12° extension to evaluate cage dislodgment in different fusion locations from top to bottom.

The minor effects of cage dislodgment were also evaluated at different cage positions through extension, lateral bending, and axial rotation. Lumbar extension can cause the cage to migrate along the positive Z direction, which is the anterior direction of the body. The results of the FEA were compared with the results of a 6° primary extension in different cage arrangements (Figure 11). An overall tendency was observed for a significantly greater cage dislodgment at lower segmental positions, corresponding to the two types of cage materials and each cage arrangement. The minimum and maximum cage dislodgments occurred in the horizontal and vertical arrangements, respectively. Moreover, the oblique arrangements of cage insertion in different fusional positions were also observed to have a low cage dislodgment. The cage dislodgments in the oblique arrangements had the same tendency as that of the horizontal cage arrangements. Further increasing the degree of extension to 12° led to similar overall tendencies of cage dislodgment in each fusion position to those for an extension of 6°, but the overall magnitude of cage dislodgment in the lumbar extension of 12° was appeared greater based on observation (Figure 12).

**Figure 13:**
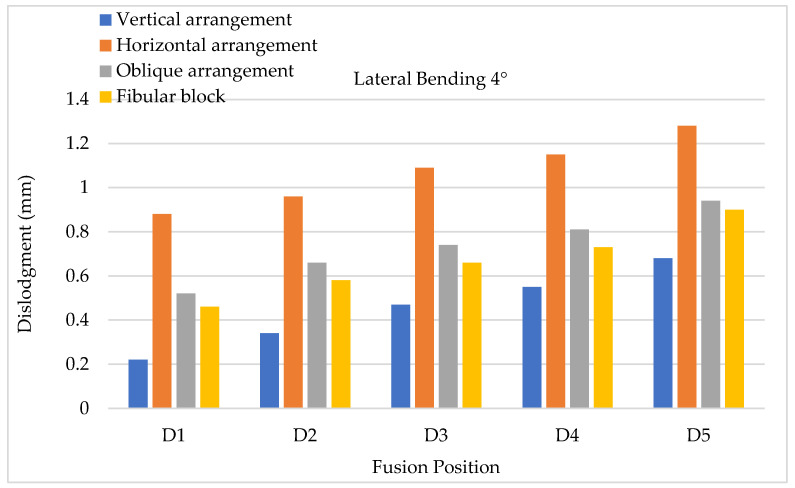
Lateral bending testing (4°) conducted to evaluate cage dislodgment in different fusion locations from top to bottom.

**Figure 14:**
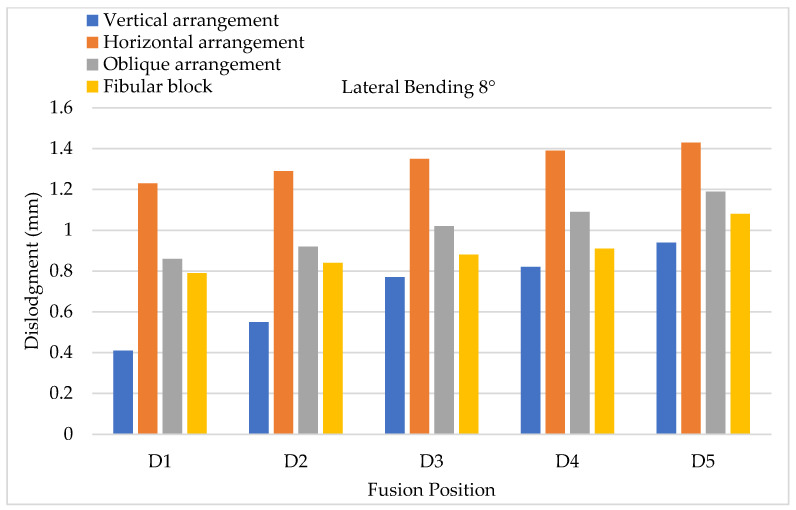
Lateral bending testing (8°) conducted to evaluate cage dislodgment in different fusion locations from top to bottom.

**Figure 15:**
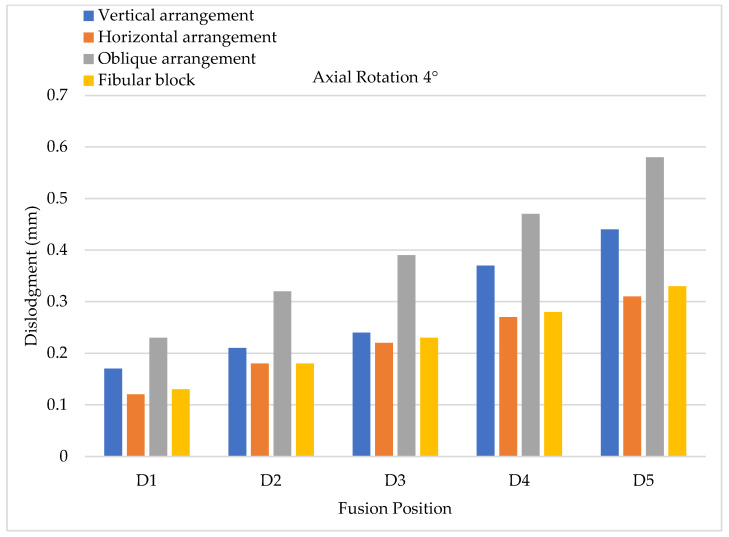
Axial rotation testing (4°) conducted to evaluate cage dislodgment in different fusion locations from top to bottom.

**Figure 16:**
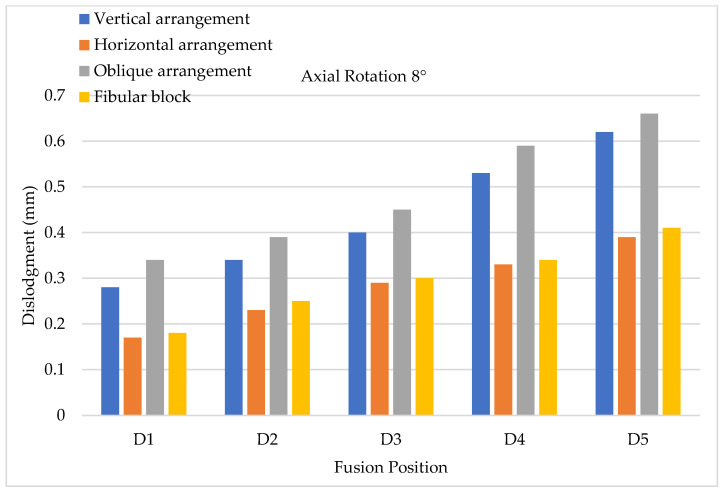
Axial rotation testing (8°) conducted to evaluate cage dislodgment in different fusion locations from top to bottom.

To evaluate cage dislodgment (centroid displacement between the period before and after cage movement) via lateral bending, the results of 4° lateral bending showed that larger cage dislodgments occurred in at a lower fusion position; hence, the maximum still occurred at the D5 position for all types of cage arrangements (Figure 13). Upon further comparing each fusion segment, the minimum and maximum cage dislodgments were detected in the vertical and horizontal arrangements, respectively. Here, 8° lateral bending showed the same tendency as 4° lateral bending, but the value of cage dislodgment was appeared greater based on observation (Figure 14). The cage dislodgment (centroid displacement between before and after cage movements) for different cage arrangements during axial rotation revealed an increasing trend when the position of the cage fusion was inferior (Figure 15). The minimum and maximum cage dislodgments were discovered in the horizontal and oblique arrangements for all of the fusion segments. The same tendency of cage dislodgment was observed when comparing the two stages of axial rotation at 4° and 8° (Figure 16). The cage dislodgment in each cage arrangement was proportional to the increase in the degree of axial rotation.

## 4. Discussion

Posterior lumbar interbody fusion has always been the most common method of lumbar segment fixation. The PLIF approach is widely considered as the optimal selection, as it avoids some kinds of vascular, visceral, and nerve damage during anterior lumbar interbody fusion surgery. PLIF can provide a better segmental stability and increase osteofusion development; however, this surgery is not always successful and presents some risk-of-failure problems. The most significant problem with PLIF is its significant risk of nerve injury. In addition, vertebral collapse, cage slippage, displacement, or implant extrusion occurs in approximately 3–10% of treated cases [45,46]. On the other hand, segmental fusion failure and cases of an artificial implant causing vertebral bone fracture have also been reported in the literature [47]. Although a fresh autologous bone graft is preferred for most lumbar interbody fusion procedures, the great quantity of sponge structures in this bone graft can provide an insufficient initial mechanical strength upon loading and immediately collapse or suffer from extruded destruction [48]. A cortical bone graft with an adequate supporting strength for the fusion segment has been developed, but the degree of fusion between the vertebrae was significantly lower. Therefore, the selection of metal or ceramic vertebral cages is the first choice for clinical surgery.

PLIF is widely used in the treatment of normal lower back pain, degenerative disc disease, recurrent disc herniation, and low-grade spondylolisthesis (grades I and II) [49]. Although the surgical outcome of PLIF has been validated, it does present some complications, such as nerve root injury, dural tear, epidural fibrosis, arachnoiditis, vertebral cage subsidence, and cage dislodgment, which are common clinical problems. Most of the complications pertaining to PLIF are related to intervertebral fusion devices, which can cause cage displacement or subsidence, showing that cage-based complications are a significant issue for further investigation. It has been considered, clinically, that the placement of a small cage in the vertebral space causes a slight amount of friction at the cage–endplate interface, leading to the posterior displacement of the inserted cage. Therefore, the shape and size of the vertebral cage should be carefully considered before surgery to avoid the backward slippage of the cage. An experienced physician should recommend that patients be evaluated via X-ray examination to determine whether they have the appropriate disc height to reduce cage backward slippage due to an incorrect cage size. On the other hand, it has been indicated in clinical studies that the placement of two cages for lumbar interbody fusion could prove more troublesome and dangerous than single-cage insertion [50]. Therefore, single-cage insertion using this technique was also considered for application in lumbar fusion surgery. Elias et al. indicated that 15% of patients experienced dural lacerations, 15% of patients experienced persistent back pain, and 15% of patients experienced radiculopathy after PLIF [51]. It is obvious that the usage of double cages has been confirmed to have more complications than the use of a single cage. A clinical report indicated that a single cage can be used for posterior lumbar interbody fusion, which is not inferior to using two cages [52]. Lumbar fusion was successful at all single-cage fusion levels and in 23/26 (88%) of all cases when reviewing all levels of fusion. Both fusion and clinical success rates were not diminished by the use of a single cage rather than the recommended two cages [53]. Therefore, the use of a single vertebral cage is in line with the norms of clinical use and does not deviate from the scope of clinical use.

In this study, we used finite element analysis to confirm that the orientation of the vertebral cage placement affected the amount of slippage between two vertebrae. In lumbar flexion movement, a horizontal placement of the cage could best prevent cage dislodgment because the maximum side length of the cage is perpendicular to the direction of the spinal movement, thus producing the maximum resistance and anti-slip characteristics for cage dislodgment. Therefore, during both lumbar flexion and extension movements, the horizontal placement of the cage could also provide the greatest benefit in terms of preventing cage dislodgment. In other words, the longer side of the cage should be immediately arranged perpendicular to the direction of spinal movement to reduce the dislodgment of the cage. According to the above inference, vertical placement of the cage will lead to the maximum amount of cage dislodgment when spinal flexion and extension are applied. Bullet-type cages are mainly inserted along the pedicle trajectory in PLIF surgery, so the cage is unable to arrange itself such that its longest side can resist the effect of cage dislodgment. Therefore, it was found that a very high proportion of patients will suffer from cage dislodgment after surgery. In severe cases of cage dislodgment in PLIF, this dislodgment can cause nerve compression complications, leading to a second operation. The horizontal placement of the cage in PLIF was confirmed to avoid and decrease cage dislodgment in lumbar flexion and extension exercises. Furthermore, this study indicated that vertical cage placement for spinal lateral bending yielded the best performance concerning the sliding resistance of the cage. On the contrary, horizontal cage placement yielded the worst performance, causing cage slippage. As for oblique cage placement, cage dislodgment was displayed between the horizontal and vertical placements of the two types of cages during flexion, extension, and lateral movements. For performing clinical PLIF surgery, it is suggested that a single cage could be inserted and then adjusted, that is, rotated and moved horizontally as far as possible, in order to effectively reduce cage dislodgment. On the other hand, the human lumbar movement is mainly supplemented by flexion and extension; hence, it is recommended that the cage be placed horizontally, which can effectively reduce cage dislodgment. More importantly, it was determined that the extent of cage dislodgment is related to different degrees of horizontal cage placement. As for the relationship between lumbar axial rotation and cage dislodgment, it was found that horizontal cage placement still yielded the smallest amount of cage slip and the oblique cage placement had the largest amount of cage slip.

The use of a single vertebral cage can not only reduce operation costs but also reduce damage to the intervertebral endplate compared with double vertebral cages, whose use more readily results in subsidence and slippage of the vertebral cage. Eck et al. [54] reported that 1.4% of patients had cage dislocation complications. This study found that the maximum displacement of cage model 1 decreased from 0.375 mm (the initial state) to 0.312 mm (the final state), and the maximum displacement of cage model 2 only decreased from 0.304 mm (the initial state) to 0.299 mm (the final state). More importantly, the single-cage model showed greater displacement in the initial state than the double-cage model, but there was a minor difference compared to cage displacement between the single and double cages in the final state. This study again supports the effectiveness of inserting parallel-placed double cages and oblique single-cage placement.

On the other hand, posterior lumbar interbody fusion (PLIF) has become a widely accepted surgical method for fixing unstable vertebral segments. It is clinically recommended to insert a bilateral vertebral cage to increase the contact area and thus improve the stability of the fused vertebral segments; however, the literature still often reports complications of vertebral cage subsidence, migration, or the compression of nerves and vessels after surgery [55]. The use of double cages increases medical costs; moreover, a wider laminectomy is required for the insertion of bilateral cages to avoid increasing the risk of bilateral nerve root injury [56]. Treatment with a single cage has been proposed in PLIF for clinical applications, and the complication rate is no higher than that when using two cages [57]. On the other hand, human cadaver experiments have shown that cage placement with an assisted pedicle fixation system in PLIF presented no differences in the stability of the fusion segments when using double parallel cages or a single cage [58]. Therefore, the usage of single or double vertebral cages can be also applied for PLIF treatment, mainly based on the experience and suggestions of clinicians with respect to selecting the cage number, constituting an important consideration in order to achieve the desirable fusion outcome and the expected stability of the fusion segment.

The PEEK cage has a hollow structure that could be subsequently filled with bone cement. There are serrated features on the upper and lower surfaces of the PEEK cage to prevent sliding, but these serrated features were omitted so that the cage sliding could be easily observed in the finite element analysis. The PEEK cage contains a long strip spacer that supports the vertebrae to maintain stability. As for the fibular vertebral cage, it is a fibular block placed in the middle area of the whole fibula. The fibula cage, which is a solid bone placed between the upper and lower vertebrae, could promote bone fusion between the vertebra and fibula. This fibular cage is fitted in an autologous bone transplantation, which can effectively improve the immediate stability of the lumbar fusion segments. Each patient has a different vertebral body size and could choose different vertebral cage dimensions. So-called poor cage dislodgment refers to the vertebral cage moving backward to compress a nerve and cause pain. Therefore, the distance between the cage end and vertebral body edge was used as the limit of permissible cage dislodgment. Going beyond this limit will lead to a high risk of nerve compression. The smaller the degree of the cage slip is, the relatively better the performance is. Cage dislodgment is linked to the vertebral body size of the patient, which determines whether it will cause adverse effects. Therefore, a better resistance to cage dislodgment results in a relatively small degree of cage slip, but this still depends on the vertebral body size of the patient. Three specific criteria were considered to determine a better ability to resist cage dislodgment in the horizontal cage arrangement: First, the distance between the cage and the vertebral body edge was determined, as a longer distance allows the cage to slide to avoid compressing the surrounding soft tissue and to avoid the vertebral margin slipping out. Second, the contact area between the cage and the vertebral body was determined, since a larger contact area can have a better slip resistance ability and cage stability. Third, the long side of the cage should be perpendicular to the sliding direction, as the long side of the cage is an obvious method for determination. These three criteria can be quantified effectively to provide a lumbar fusion reconstruction as a reference for selecting a cage arrangement.

Comparisons of the cage placement orientation for fusion surgery using a single cage revealed a notable relationship between cage arrangement and cage dislodgment according to finite element analysis. The results for the bullet-shaped PEEK cage showed that the horizontal placement of the cage could effectively reduce cage dislodgment when performing single-cage insertion. Horizontal cage placement has an excellent advantage with regard to preventing slippage, and this result has the same trend as shown in previous studies [59,60]. The efficacy of preventing slippage of the cage model using the fibular block was not remarkable. A fibular block is generally used in fusion surgery to improve the efficacy of bone fusion, but it is not significantly beneficial for reducing subsequent cage dislodgment [61]. It is speculated that the finite element model does not reveal the bonding contact relationship between the fibula and the end plate of the vertebrae after bone fusion, so the fibula block can slide after lumbar spine movements and the cage of the fibula block does not reduce dislodgment to a greater degree than the PEEK cage. Notably, the finite element analysis in this study only analyzed and compared the shape and orientation of the cage and did not investigate the influence of the cage after bone fusion. Regarding the effects of bone fusion between the cage and the vertebrae, a subsequent finite element analysis will be planned and explored to provide a better understanding of the long-term performance of the fibula cage and the PEEK cage after surgery. The tests of cage dislodgment with different orientations and materials showed that the magnitudes of the cage dislodgment were successfully reflected through finite element analysis. Moreover, the largest amount of cage dislodgment was confirmed to occur during lumbar flexion movement, and this outcome has the same trend as shown in the clinical literature [21,62]. This study verified that single-cage placement in PLIF using horizontal positioning is ideal for preventing cage dislodgment. A second option is also recommended—that is, oblique cage placement—to reduce cage dislodgment in PLIF caused by frequent flexion movement if a horizontal cage cannot be placed.

Finite element analysis (FEA) has been applied to investigate spinal biomechanics for more than 30 years. FEA results have shown that the variances in stress distributions and displacement locations in the vertebrae and cage are caused by different cage numbers and placements. A previous study by Chiang et al. [32] was chosen to compare with our FEA results. One of the aims of Chiang’s study was to evaluate dislodgment differences between one and two cages in the L4–L5 segment. Single and double cages were arranged in 45 oblique and vertical placements, respectively. The extent of the cage dislodgment of a single cage was slightly higher than that of two cages. The maximum and second highest cage dislodgments in a single cage were detected in rotation (0.25 mm) and flexion (0.03 mm), respectively. The results of cage dislodgments in Chiang’s study are clearly lower than those in the present study. These cage dislodgments (≤0.25 mm) were not significant for the four types of lumbar movements after surgery. The maximum cage dislodgment (0.25 mm) in Chiang’s FEA is clearly too small to cause nerve compression after surgery. This was not compatible with the clinical results. Additionally, the clinical criterion of the case dislodgment should be based on whether the cage has touched the nerve and caused pain. We infer some reasons for the small degree of cage dislodgment in Chiang’s study. First, they used a very small movement angle at L4–L5 (approximately in the range of 0.54° to 1.41°). Second, they used a larger length of the cage body (30 mm) and a larger contact area between the cage and the vertebrae (191.5 mm^2^). Third, they selected a small preload application (150 N). Fourth, they meshed a few elements in the lumbar spine model (16,808). Fifth, they assumed a simplified geometry of the regional lumbar model (rough geometry) and a simplified definition of the surface-to-surface contact element between the cage and the vertebrae (the friction coefficient was set to 0.1). In particular, the influence of the movement angle was the largest since there was almost no movement. In conclusion, it is difficult to compare different studies due to different settings, such as differences in model structures, material parameters, boundary conditions, load conditions, and parameter combinations.

A comparison of cage dislodgment from human experiments and FEA is very important to confirm whether the result of the finite element analysis is valid. Therefore, further validation of this study was conducted by first comparing the influence of the fusion position to a previous study, which analyzed the cage-retropulsion-associated risk factors retrospectively [63]. The same tendency of the lower portion in the fusion segment to induce a higher magnitude of cage dislodgment under lumbar movements was evidenced. It was easier to produce cage slip in the lower fusion segment. The simulation analysis produced similar results to those from an actual patient following assessment. Hence, the results of the present study appeared to confirm the same trend as Zhang et al.’s clinical retrospective study.

Furthermore, finite element analysis has also been used to predict adjacent soft tissue degradation and the stress failure trends of fixed devices after fusion surgery [64,65]. To investigate the effect of cage dislodgment with the placement of a single vertebral cage, a finite element model of the lumbar spine, including discs and ligaments, was standardized and matched, as much as possible, with the structure of a real fused vertebral segment of a fusion patient with a postoperative vertebral cage position in PLIF. The standardized numerical lumbar model can provide valid data after analysis and can effectively be used to investigate the influence of slippage in different placement orientations of the cage. As shown in this study, the cage made from PEEK material more appropriately reflected the material properties of vertebral bone. The fusion segment in the posterior vertebral area was also removed precisely following the clinical protocol in order to more accurately calculate the stress distribution and evaluate the performance of cage dislodgment. This study investigated the influence of different cage placement orientations on cage dislodgment so as to clarify the best and worst cage placements of PLIF using a single cage. Additionally, this study was also performed to compare the effects of cage dislodgment for two types of PEEK materials and a fibular block.

Cage dislodgment in patients receiving fusion surgery is an important evaluation index for understanding the cage’s stability in the fusion segment. A larger cage dislodgment could cause a failure, leading to nerve compression. Therefore, it is important to determine what kinds of cage arrangements should be selected in PLIF. Horizontal placements of the PEEK cage effectively reduce the cage dislodgment in patients by a larger amount to avoid nerve compression for a sustained period. The results of this study evidenced that different cage arrangements could influence the degree of cage dislodgment, particularly the horizontal placement of the PEEK cage in flexion. The dislodgment of the fibular cage was discovered to have a larger risk compared with that of the PEEK cage. The reasons for this are a smaller contact area and an assumption that no bone fusion occurred. This study provides potential information about the relationship between cage arrangement and cage dislodgment in PLIF for clinical surgical applications and as a reference. Therefore, the horizontal cage arrangement is suggested for PLIF to avoid nerve compression or soft tissue rupture.

The results of this study were obtained based upon finite element analysis; hence, several limitations are listed below. First, the assumption that the materials are homogeneous and isotropic with respect to real human spinal materials may be incorrect, and directional loading conditions were not reflected for real lumbar muscular forces. The assumption of homogeneous and isotropic material properties in the FEA did not affect the cage dislodgment. As for muscle forces, these were not applied in this study due to the assumption of torque, which was mainly made in accordance with previous studies. In other words, the loading condition of the muscular forces in the lumbar was replaced by torque for investigating different movements in the FEA. Second, these models did not include bone cement in the FE model, which only investigated the relationship between vertebral cage placement arrangements and backward dislodgment. Cage migration, which was discovered by using bone cement, was detected in patients after PLIF in previous studies [35,66]. Obviously, cage dislodgment always occurs with and without using bone cement after fusion surgery. Hence, the limitation of the lack of cement in the spinal fusion model in this study could be neglected to allow for an investigation of the cage dislodgment. Therefore, the results regarding vertebral cage dislodgment may be enhanced in terms of the magnitude of cage displacement. Third, this study did not further investigate stress distributions on the vertebral body or endplate for different cage placement arrangements. The relationship between the stress distribution and cage dislodgment was not significant in the investigation of cage slippage. Furthermore, as for the influence of the vertebral geometry, which was determined by means of scanning a replica, the replica was a commercial product with an actual geometry and anatomical features in the regions of the endplate surface, spinal process, and vertebral shape. Therefore, the problem of an imprecise lumbar model was avoided in this FEA study. Finally, the finite element models in the present study did not account for the nucleus pulposus within the disc when evaluating cage effects. This is because the relationship between the material of the nucleus pulposus and the behavior of cage dislodgment is not very significant.

## 5. Conclusions

The FEA results confirmed that the different placements, such as vertical, horizontal, and oblique arrangements, of the PEEK cage had different effects on the extent of cage dislodgment. The flexion movement of the lumbar spine caused the largest dislodgment and was the main movement causing postoperative failure. Evidently, a horizontal arrangement of the PEEK cage is better for preventing cage dislodgment between two vertebrae in the lumbar fusion segment. A horizontal arrangement of the PEEK cage can lead to a better performance regarding cage dislodgment in terms of flexion, lateral bending, and axial rotation. It is suggested that, if a single cage is used, it should be placed horizontally to reduce the risk of cage dislodgment for lumbar fusion patients. In addition, compared with the PEEK and fibular cages, the bullet-type PEEK cage was shown to have a better anti-slippage ability. The fibular cage may be influenced by the upper and lower contact shapes and areas, which ineffectively reduce cage dislodgment. As for the influence of the location of the fusion segment, a lower fusion position will produce a greater amount of cage dislodgment. The extent of cage dislodgment in the lower part of the fusion segment will be higher than that in the upper fusion segment, and this phenomenon shows the same trend in all lumbar movements.

## Figures and Tables

**Table 2:** Comparison of mean values of ROM in intact spine motions ranging from L1 to the sacrum.

Intact Spine Motion	Flexion	Extension	Left Lateral Bending	Left Lateral Bending	Left Axial Rotation	Right Axial Rotation
Present FE model	52.6	16.5	20.2	18.1	8.1	8.8
Pearcy (1985) [42]	51.0	16.0	18.0	17.0	5.0	4.0
Lee and Wong (2002) [43]	58.1	15.6	21.3	19.9	7.6	9.8
Lee et al. (2003) [44]	48.6	18.7	16.3	16.3	8.9	8.4

## Data Availability

The datasets used and/or analyzed in the current study are available from the corresponding author on reasonable request.
